# Tackling the Complexity of the Exposome: Considerations from the Gunma University Initiative for Advanced Research (GIAR) Exposome Symposium

**DOI:** 10.3390/metabo9060106

**Published:** 2019-06-06

**Authors:** Pei Zhang, Manish Arora, Romanas Chaleckis, Tomohiko Isobe, Mohit Jain, Isabel Meister, Erik Melén, Matthew Perzanowski, Federico Torta, Markus R. Wenk, Craig E. Wheelock

**Affiliations:** 1 Gunma University Initiative for Advanced Research (GIAR), Gunma University, Maebashi 371-8510, Japan; peizhang@gunma-u.ac.jp (P.Z.); romcha@gunma-u.ac.jp (R.C.); isabel.meister@gunma-u.ac.jp (I.M.); 2 Division of Physiological Chemistry 2, Department of Medical Biochemistry and Biophysics, Karolinska Institutet, Solna 171 77, Sweden; 3 Senator Frank R Lautenberg Environmental Health Sciences Laboratory, Department of Environmental Medicine and Public Health, Division of Environmental Health, Icahn School of Medicine at Mount Sinai, New York, NY 10029, USA; manish.arora@mssm.edu; 4 National Center for the Japan Environment and Children’s Study, National Institute for Environmental Sciences, Tsukuba 305-0053, Japan; isobe.tomohiko@nies.go.jp; 5 Department of Medicine, University of California, San Diego, La Jolla, CA 92093, USA; mjain@ucsd.edu; 6 Department of Pharmacology, University of California, San Diego, CA 92093, USA; 7 Institute of Environmental Medicine, Karolinska Institutet, Solna 171 77, Sweden; Erik.Melen@ki.se; 8 Department of Clinical Sciences and Education, Karolinska Institutet and Sachs’ Children’s Hospital, Stockholm 118 83, Sweden; 9 Department of Environmental Health Sciences, Mailman School of Public Health, Columbia University, New York, NY 10027, USA; mp2217@cumc.columbia.edu; 10 Department of Biochemistry, YLL School of Medicine, National University of Singapore, Singapore 117597, Singapore; bchfdtt@nus.edu.sg (F.T.); bchmrw@nus.edu.sg (M.R.W.)

**Keywords:** exposome, metabolomics, exposure, epigenetics, environment

## Abstract

The attempt to describe complex diseases by solely genetic determination has not been successful. There is increasing recognition that the development of disease is often a consequence of interactions between multiple genetic and environmental factors. To date, much of the research on environmental determinants of disease has focused on single exposures generally measured at a single time point. In order to address this limitation, the concept of the exposome has been introduced as a comprehensive approach, studying the full complement of environmental exposures from conception onwards. However, exposures are vast, dynamic, and diverse, and only a small proportion can be reasonably measured due to limitations in technology and feasibility. In addition, the interplay between genes and exposure as well as between different exposures is complicated and multifaceted, which leads to difficulties in linking disease or health outcomes with exposures. The large numbers of collected samples require well-designed logistics. Furthermore, the immense data sets generated from exposome studies require a significant computational investment for both data analysis and data storage. This report summarizes discussions during an international exposome symposium held at Gunma University in Japan regarding the concept of the exposome, challenges in exposome research, and future perspectives in the field.

## 1. Introduction

Since the human genome was sequenced, an extreme effort has been placed into mapping the role of genes in the onset of disease. It was expected that we would be able to explain the cause of disease, as well as understand the genetic basis of health. However, we have found that while the genetic contribution to different diseases varies, nongenetic factors have much greater attributable risks, often in the range of 80 to 90% for complex diseases [1]. This dominance of nongenetic components emphasizes the role of the environment in the risk of the development of chronic disease. In 2010, there were >50 million global deaths, of which ~50% could be attributed to a small set of exposures consisting primarily of particulate air pollution, smoking, and diet [2]. This suggests that if we can identify relevant exposures, we can potentially intervene to reduce the burden of these chronic diseases.

We now know that adverse gene–environment interactions probably influence most chronic diseases, including respiratory disease, allergy, and cancer [3]. However, the tools for quantitative assessment of exposures, based on measurements of chemicals in air, water, food, and the human body, are still limited in their capacity to detect multiple exposures simultaneously. This lack of comprehensive profiling methods to assess exposure has resulted in a need to rely upon self-reported information supplemented with population level data from multiple sources (e.g., demographics, census data, green space surveys, and GIS approaches) to categorize exposures. These self-reports, with the general exceptions of alcohol consumption and smoking, are unreliable predictors of long-term exposure levels and are poorly suited for assessing exposome interactions. While the extensive investment in genomics has successfully illuminated the genetic determinants of diseases, we are still in the dark ages when it comes to quantifying exposures.

**Figure 1:**
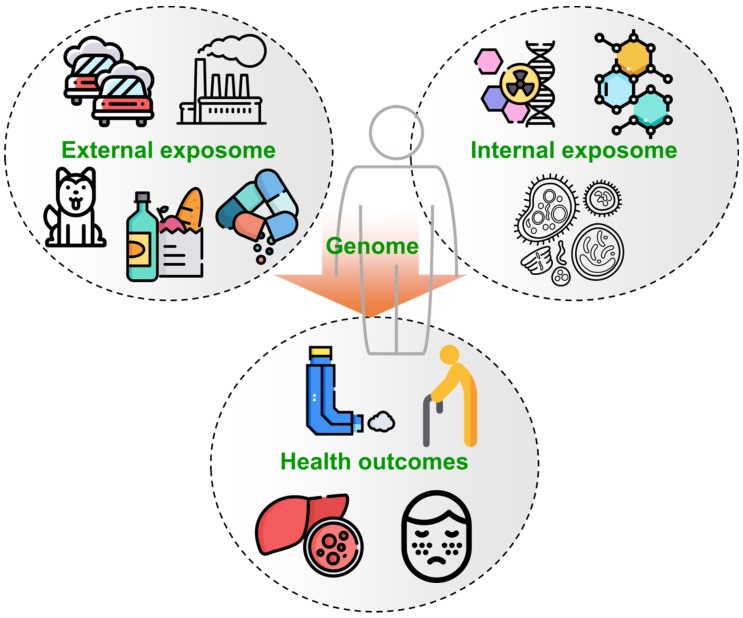
The concept of the exposome. The human exposome is often divided into the external exposome (e.g., air, diet, and social factors) and internal exposome (biological responses to exposure). Interactions between exposure and genetic factors can lead to disease or adverse health outcomes (e.g., aging, allergy, asthma, and cancer). Icons are designed by Freepik.

In recognition of this disparity in current knowledge on the health associations of the interactions between genes and environmental exposures, the term exposome has been proposed to represent all environmental exposures (including those from diet, behavior, and endogenous sources) from conception onward [4,5] (Figure 1). The exposome encompasses all nongenetic influences, and has become of critical relevance to understanding disease etiology [4], with particular interest in examining the pregnancy exposome and birth cohorts in disease presentation [6]. A few initiatives have begun, including EXPOsOMICs [7,8], HEALS (Health and Environment-wide Associations based on Large population Surveys) [9], and HELIX (the Human Early-life Exposome) [10] supported by the EU; CHEAR (the Children’s Health Exposure Analysis Resource) [11] and HERCULES (The Emory Health and Exposome Research Center: Understanding Lifetime Exposures) funded by the National Institute of Environmental Health Sciences (NIEHS) in the US; JECS (the Japan Environment and Children’s Study) launched in Japan [12]; and the I3CARE International Exposome Center collaboration between the Chinese University of Hong Kong, the University of Utrecht and the University of Toronto. However, extensive efforts are still needed to develop the methodologies to capture the exposome. An international symposium focusing on the exposome was held at Gunma University, Japan, on 23 October 2018. All of the authors on this report attended the meeting and a list of the talks is provided in Figure S1. Based upon the current status of the exposome field, this report summarizes opinions and perspectives with respect to study design, exposure measurement, sample collection and storage and future outlooks from discussions during the symposium.

## 2. What is the Exposome?

The exposome can be defined as the total sum of all exposures from conception onwards [13,14]. The concept of the exposome was introduced as the environmental counterpart to the genome in order to establish the role of environmental exposures on a given genetic background. An exposure comes from the external environment (e.g., environment, lifestyle, stress, diet, and noise) and includes our internal biological processes (e.g., metabolism). The exposome is therefore often recognized as being comprised of the external and internal exposome (Figure 1) [15]. The external exposome refers to the full set of external exposures and experiences potentially contributing to disease or health outcomes (see Table 1 for some examples). The internal exposome includes biological responses to the external environment, interactions with exogenous compounds (e.g., interactions between diet and the gut microbiome [16,17]) and exogenous compounds entering the internal environment via diet, smoking, water, air, etc.

## 3. How to Perform an Exposome Study?

Implementing the exposome concept to explain the causalities of chronic disease poses multiple challenges [18]. Herein, we discuss four basic questions: (1) Which variables should be included in the study design? (2) How to develop methods to comprehensively assess all exposures from both external and internal sources? (3) Which sample types can be collected and how to effectively manage large sample numbers? (4) How to dissect the associations between exposome data and health end points?

### 3.1. Study Design

As the field of exposome science continues to develop, a fundamental question is how to perform an exposome study. There are no clear methodologies and only a few examples in the literature. To date, three overall strategies have been applied. The first one is called “bottom-up”, in which external exposures are comprehensively measured and then linked to disease or health outcomes [6,19]. The second strategy is “top-down”, in which omics technologies (e.g., metabolomics, adductomics, and epigenomics) are applied to identify biomarkers of exposures and new causes of or associations with disease [20,21]. The last approach is “meet-in-the-middle”, in which the association between exposures and disease or biomarkers of exposures are initially investigated and then the relationship between disease and biomarkers is assessed. No matter which strategy is applied, an exposome study should be assumption-directed. For example, if the study is designed to explore the role of environmental factors on asthma, information on all exposures that could potentially affect airway inflammation should be collected [22]. However, this will come at the cost of not identifying rare exposures that are not yet known to correlate with a given disease; whereas, if the study aims to discover effects of exposures on unexpected health outcomes, it is desirable to include all measurable exposures in a given time period. However, this approach is not feasible and therefore some basic assumptions must be made in the initial experimental design in terms of which exposure variables are most relevant to the study at hand. The epidemiology field is well versed in designing these complex studies, with multiple options depending upon the study design (e.g., cross-sectional, longitudinal, cohort, and case–control). The different study designs can also complement one another, and there may be advantages in integrating them to gain an improved understanding of the effects of exposure.

One key challenge is to identify cause and effect in exposome association studies. While it is not possible to fully assign cause and effect in the design of an exposome study, some concepts can aid in interpreting findings. Due to the observational and cross-sectional study design of most studies conducted to date, there is often a temporal discordance between exposure and observed effect. It is therefore difficult to understand the effect of potential exposures from single spot samples. Exposome-based studies therefore benefit from longitudinal sampling, particular during critical life stages [23]. Accordingly, study design needs to incorporate multiple longitudinal time periods as well as temporal variability [24]. Many exposome studies employ birth cohorts, which enables studies to monitor the health trajectory of a population over time (including critical periods early in life) and investigation of the effects of multiple exposures [6].

The potential scale of exposome work can be massive by definition and is often beyond the ability of a single research group, requiring collaborations. It is not necessary to initiate an exposome study from scratch, and finished or ongoing associated projects can be leveraged and re-analyzed to suit exposome research aims. For example, HELIX combines six established and ongoing longitudinal population-based birth cohort studies in six European countries, serving as a good example of integrating existing resources [19].

Nonetheless, there remain challenges of study design and exposure reconstruction. First, for rare and low frequency disorders, especially those with long latency periods, establishing prospective studies is not always feasible as it would require very large samples sizes and many years of follow-up to obtain sufficient cases. For example, amyotrophic lateral sclerosis (ALS) is a disease that occurs with a frequency of 2 per 100,000 and is most often diagnosed in the fifth decade of life [25]. To study the early life environmental determinants of this neurodegenerative disorder, a sample size in excess of a million participants who are studied over decades would be needed in a prospective cohort design. A second major challenge is to retrospectively measure fetal exposure, which is a critical period of development. Many extant studies do not have biobanked samples of umbilical cord blood. An alternative to this approach is the use of naturally shed deciduous teeth or permanent adult teeth. Deciduous teeth commence development prenatally and grow in an incremental pattern (akin to growth rings in trees). Using beam-based methods, such as laser ablation, it is now possible to reconstruct past exposures (e.g., metals and organic chemicals) at fine temporal resolution, including over the fetal developmental period [26,27]. However, this approach requires significant expertise in dental tissue biology and has relatively complex sample preparation methods. For the external environment, there exist methods to reconstruct particulate air pollution, ambient temperature, green-space, light-at-night, and other measures using satellite-based monitoring [28,29,30]. These tools allow fine temporal scale reconstruction of historical exposure that may be added to existing studies.

An exposome study can also be fulfilled without extensively measuring exposures in large-scale biological samples. For example, efforts have been made to dissect the relationship between the genome and environment in large data streams such as electronic health records and epidemiological cohorts using tools of bioinformatics [31,32]. In a recent study [33], a large health insurance dataset was analyzed to assess the genetic and environmental contributions of 560 disease-related phenotypes in 56,396 twin pairs and 724,513 sibling pairs. The contribution of specific environmental risk factors like socioeconomic status and air pollution in phenotype were also estimated. Notably, this type of study requires massive data sets, vast experience in data analysis as well as comprehensive knowledge in genetics and epidemiology.

### 3.2. Which Exposures to Measure

Due to the comprehensive nature of the exposome, it is essentially not possible to measure the full complement of exposures. It then becomes a question of which exposures are the most informative as well as most realistic to obtain. A nonexclusive list of potential exposome categories and examples is shown in Table 1. External exposures such as climate and air quality can be acquired from local meteorological stations and environment databases (e.g., Global Environmental Database, Japan). The use of GIS (Geographical Intelligent Software) is also particularly useful for acquiring this type of data. Personal life style and behavior as well as social factors are typically obtained by questionnaires or self-reports. Smartphone-based personal wearable devices can monitor basic functions including physical activity, heart rate, sleep quality, food nutrition, etc. Silicone bands that are capable of capturing airborne or skin contact exposures including pesticides and flame retardants are now available (MyExposome, Inc., Corvallis, OR, USA) [34,35]. More professional devices have also been invented, for example a modified MicroPEM™ (RTI International, Research Triangle Park, NC, USA), which includes an infrared laser nephelometer, a PTFE (polytetrafluoroethylene) or PES (polyethersulfone) filter, and a cartridge filled with zeolite adsorbent beads that can capture hundreds of airborne biological and chemical exposures [36].

Current exposome studies mostly focus on measurable environmental exposures (e.g., air pollution, smoke, and occupational exposure) due to knowledge and technology limitations. However, there is a need to have as comprehensive of a measurement as possible. Measurable internal biological changes can serve as good indicators of external exposures, for example, telomere length is a master integrator of stressors that result from a variety of lifestyle and behavioral factors [37]. More comprehensively, omics technologies are recognized as promising approaches for obtaining biomarkers of exposure, biomarkers of effect, and biomarkers of susceptibility. In particular, it would be useful to identify biomarkers of susceptibility in order to identify at-risk or sensitive populations for certain environmental exposures. While top-down measurement of exposome signals through measurement of the global biological response using omics technologies addresses the issue to some extent, the current analytical technology is not sufficiently sensitive or flexible to capture and identify the components of the exposome in a single method [38,39]. Accordingly, current approaches will have to focus on specific chemical classes and integrate multi-platform data to generate more informative datasets.

### 3.3. Sample Collection and Management

The most common and readily available biological samples include blood and urine. Additional matrices of interest include feces, hair, sputum [40], and breast milk. Another approach that has been particularly successful is the use of teeth [41,42,43]. As mentioned earlier, the incremental and archival nature of teeth is an attractive option for obtaining detailed information on exposure timing. For example, a tooth-matrix biomarker method identified that metal toxicant uptake and essential element deficiency increased the risk of autism spectrum disorder in children [44].

Exposome studies generally involve large cohorts in order to have sufficient statistical power to detect meaningful effect sizes. For example, the JECS study is only halfway through its targeted completion, and has already amassed >5,000,000 individual sample tubes collected from >250,000 participants (>100,000 expecting mothers, >100,000 children, and >50,000 fathers). Exposure to environmental factors are being assessed by chemical analyses of biospecimens (e.g., blood, cord blood, urine, breast milk, and hair), household environment measurements, and computational simulations using monitoring data (e.g., ambient air quality monitoring), as well as questionnaires. The logistics of managing this many samples should not be underestimated and protocols with respect to sample collection, pretreatment, transportation, aliquoting, labeling, and storage should be decided early in the study design [12]. Biological samples should be aliquoted into small volumes to minimize potential effects from repeated freeze–thaw cycles [45]. Another important component is the question of sample integrity during storage. Some cohort studies may include samples that are decades old and there is little information on the stability of materials overtime. It would be particularly valuable to have molecular markers of sample viability, and there have been some initial efforts in this area [46,47].

### 3.4. Exposome Data Analysis

The basic concept of the exposome is to determine how environmental factors affect human health. Therefore, when it comes to data analysis, it is vital to link exposures to disease or health [48]. However, prior to data analysis, obtained exposures (variants) need to be preprocessed or cleaned (e.g., address missing values, classify the exposures into categories, calculate the correlations between and within categories, narrow down the variant dimensions by excluding co-occurring exposures, and data transformation). These efforts are nontrivial, and require a clear data analysis plan prior to study initiation.

While GWAS has been extensively used to identify genetic variants associated with disease, the concept of exposome-wide association study (EWAS) is still relatively new [9,49]. EWAS borrows the methods of GWAS to identify environmental factors that are associated with specific phenotypes. The logical next step in these analyses is the concept of genome-wide by environment interaction studies (GWEIS) in large-scale settings [50]. GWEIS approaches have been applied in various studies to investigate if there are specific variants associated with disease risk following exposure, or to identify relevant disease mechanisms. For example, novel loci for childhood asthma in relation to traffic-related air pollution exposure have been identified in recent GWEIS [51], as well as loci for respiratory symptoms in relation to occupational exposures [52] and for serum lipids in relation to smoking [53]. However, appropriate statistical models to conduct genome-wide studies in a true exposome setting have yet to be developed and well-powered studies to be performed. Likewise, epigenome-wide studies have been conducted for single environmental exposures linking, for example, maternal tobacco smoke during pregnancy [54] and prenatal air pollution exposure [55] to newborn DNA methylation changes, but no comprehensive exposome–epigenetics study has been published to date. Yet, these epigenetics studies highlight the complex interplay between environmental exposures and our genetic setup.

An interesting method to present exposure interactions is the exposome interaction network, which combines human- and environment-related exposures. Data analysis was designed to examine the intersection of interacting ecosystems (e.g., human-related bacteria, fungi, and arthropods). An exposome cloud was generated by querying species-interaction databases based on all measured biotic exposures. The number of nodes/edges/average interactions in the cloud provides an overview of personal lifestyles and work–home routines [36]. This method represents a realistic approach to interrogate and analyze this large body of data in a meaningful fashion that is accessible for the interested reader.

## 4. Future Perspectives

There is a need to define the composition of the exposome in order to assist in experimental design and to move the field forward. A number of efforts have already been made to develop exposome databases [56,57,58]. For example, metabolomics databases have begun to focus on exogenous chemicals and METLIN has expanded its library to include >700,000 chemicals [59]. In addition, there are decades of studies performed in the environmental field on a compound-by-compound basis. Accordingly, a vast, scattered exposure database already exists, representing an opportunity to collect a large body of information on the effects of exposure. There are also significant data available from workplace and occupational exposures [60]. In addition, multiple groups have performed targeted environmental exposures [61,62,63] as well as work performed in model organisms [64]. However, in order to make effective use of these resources, there is a need for extensive data sharing and for platforms capable of processing and analyzing large-scale data.

The standard approach to an exposome study generally involves large numbers of individuals in order to obtain reliable exposure-phenotype interactions. However, it is also possible to focus on fewer participants for a longer period (i.e., longitudinal study). In a recent study, 15 individuals were followed for up to 890 days to discover the diversity and dynamics of the exposome and its potential impact on human health [36]. Apart from revealing the casualties of disease, the exposome might also be useful for providing individual feedback (e.g., health status, local environment and quality), heritability prediction, and identifying individuals at risk. The exposome will most likely become an important component of personalized health care, together with genetic and other omics data, in order to establish which exposures may result in adverse health effects for individuals. The combination of genetic determinants with exposure data may be able to identify susceptible subpopulations that have higher risk levels of disease development in association with specific exposures. It is likely that the near future of health care will incorporate a combination of personal, home, and work space monitoring to measure exposure profiles, which will be correlated with individual susceptibility to disease and ultimately assist in maintaining health.

## 5. Concluding Remarks

There is a strong link between environmental exposure and disease onset; however, few efforts have been made to determine the total human exposure from our environment. The advent of the exposome is an attempt to understand the important, but complex nature of the interactions between environmental exposures, genetics, and health. An exposome study must incorporate multiple potential confounders including population vs. individual variation, age effects, seasonal effects, spatial effects, etc. In the future, it will be important to systematically expand the depth and breadth of our exposure knowledge. More importantly, the development of exposome science will require extraordinary efforts in many disciplines (environmental science, analytical chemistry, bioinformatics, genetics, systems biology, and epidemiology). Scientists from these fields, policy-makers, and funding agencies need to harmonize their approach in order to tackle the complexity of the exposome, which offers nothing less than the potential to understand the etiology of heterogeneous diseases.

## Figures and Tables

**Table 1:** Relevant exposures for inclusion in exposome studies.

Exposure Group	Exposure
**External**	
- Meteorology	Climate change, temperature, humidity, wind, atmospheric pressure
- Outdoor exposures	NO_2_, SO_2_, CO, O_3_, VOCs, PM, radiation, UV, traffic, pollen
- Built environment	Population density, building density, facilities, green space, walkability, neighborhood safety, accessibility to resources (e.g., hospitals, bus stations), noise
- Home environment	VOCs, PM, NO_2_, CO, aldehydes, metals, plasticizers, dust, pets, pests, allergen (e.g., house dust mites), mold, fungi, microbes, endotoxin
- Personal behavior	Diets, physical activity, tobacco smoke, alcohol, drugs, sleep, sex, cosmetics
- Social economic factors	Social factors, education, economy, psychological and mental stress
- Food and water contaminants	Fertilizers, metals, pesticides, plasticizers, DBPs, PCBs, flame retardants, PFASs
- Medications	Medicines, surgeries
- Occupational exposures	Chemicals, dust, metals, virus, animal proteins, plants, heat/cold stress
**Internal**	
	Primary external exposures and associated metabolites, epigenetic (e.g., methylations, histone modifications), microbiome/metabolome/proteome/transcriptome/genome changes, etc.

Abbreviations: VOCs: volatile organic compounds; PM: particulate matter; PCB: polychlorinated biphenyl; NO_2_: nitrogen dioxide; UV: ultraviolet; CO: carbon oxide; O_3_: ozone; SO_2_: sulfur dioxide; DBPs: (water) disinfection by-products; PFASs: per- and polyfluoroalkyl substances.

## Supplementary Materials

The following are available online at https://www.mdpi.com/2218-1989/9/6/106/s1, Figure S1: Program of the 5th International Symposium of the Gunma University Initiative for Advanced Research (GIAR).
