# Supplementary material for: Tackling the Complexity of the Exposome: Considerations from the Gunma University Initiative for Advanced Research (GIAR) Exposome Symposium
Source: Metabolites. 2019 Jun 6;9(6):106. doi: 10.3390/metabo9060106 (PMC6631702; doi:10.3390/metabo9060106)
Supplement: Supplementary file 1 [file metabolites-09-00106-s001.pdf]

GUNMA UNIVERSITY

The 5th International Symposium of Gunma University Initiative for Advanced Research (GIAR)

Date Tuesday, October 23rd, 2018
13:00～19:00 (OPEN 12:45)

Venue Tojo Hall
@Gunma University Showa Campus

WELCOME ADDRESS

13:00 ～ 13:05 Hiroshi Hiratsuka (President of Gunma University)

[SESSION 1] Frontiers in Metabolome and Proteome Research

13:05 ～ 13:10 Session overview (Hideru Obinata, GIAR)

13:10 ～ 13:40 "^{13}C-Metabolic flux analysis of drug-induced rewiring of cancer cell metabolism"
Fumio Matsuda (Graduate School of Information Science and Technology, Osaka University)

13:40 ～ 14:10 "Development and application of mass spectrometry-based lipidomics strategy in basic and clinical research"
Yoshihiro Kita (Graduate School of Medicine, The University of Tokyo)

14:10 ～ 14:40 "Capillary electrophoresis-mass spectrometry (CE-MS)-based metabolomics and its clinical application"
Akiyoshi Hirayama (Institute for Advanced Biosciences, Keio University)

14:40 ～ 15:10 "Recent progresses of quantitative proteomics in clinical research"
Sumio Ohtsuki (Faculty of Life Sciences, Kumamoto University)

15:10 ～ 15:30 COFFEE BREAK

[SESSION 2] The Exposome: Crossroads in Metabolism, Disease and the Environment

15:30 ～ 15:35 Session overview (Craig Wheelock, Karolinska Institutet/GIAR)

15:35 ～ 16:05 "An update on epigenetics, environmental exposure and respiratory disease"
Erik Melén (Institute of Environmental Medicine, Karolinska Institutet)

16:05 ～ 16:35 "Neighborhood environmental determinants of asthma morbidity"
Matthew Perzanowski (Mailman School of Public Health Columbia University)

16:35 ～ 17:05 "Mapping human chemical diversity and the non-genetic landscape of disease"
Mohit Jain (School of Medicine, University of California San Diego)

17:05 ～ 17:35 "Early life environment and neurodevelopmental trajectories"
Manish Arora (Department of Environmental Medicine and Public Health, Icahn School of Medicine at Mount Sinai)

17:35 ～ 17:50 SHORT BREAK

INTRODUCTION OF A NEW INTERNATIONAL OPEN LABORATORY AT GIAR

17:50 ～ 18:05 "Design and preparation of innovative functionalizable silsesquioxanes as building blocks for new nano-sized supported catalysts"
Yujia Liu (International Open Laboratory-ENSCM Lab, GIAR)

SPECIAL LECTURE

18:05 ～ 18:55 "Translation of lipidomic technologies towards quantification of blood lipids and their natural variations"
Markus Wenk (Yong Loo Lin School of Medicine, National University of Singapore)

CLOSING REMARK

18:55 ～ 19:00 Takashi Minegishi（GIAR chairperson / Vice President of Gunma University）

Registration required to attend the reception from 19:30 at Cinema restaurant
Please send an email to kk-kensui4@jimu.gunma-u.ac.jp by Oct. 16th
